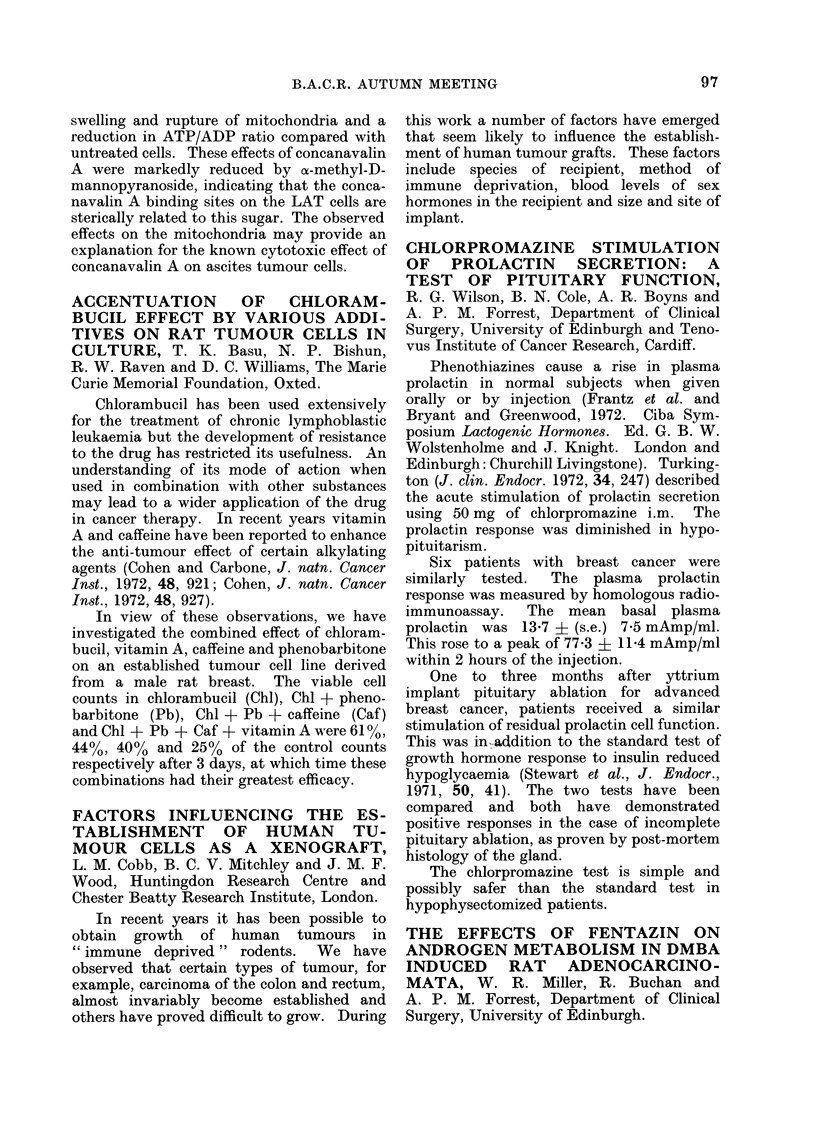# Proceedings: Factors influencing the establishment of human tumour cells as a xenograft.

**DOI:** 10.1038/bjc.1974.31

**Published:** 1974-01

**Authors:** L. M. Cobb, B. C. Mitchley, J. M. Wood


**FACTORS INFLUENCING THE ESTABLISHMENT OF HUMAN TUMOUR CELLS AS A XENOGRAFT,** L. M. Cobb, B. C. V. Mitchley and J. M. F. Wood, Huntingdon Research Centre and Chester Beatty Research Institute, London.

In recent years it has been possible to obtain growth of human tumours in "immune deprived" rodents. We have observed that certain types of tumour, for example, carcinoma of the colon and rectum, almost invariably become established and others have proved difficult to grow. During this work a number of factors have emerged that seem likely to influence the establishment of human tumour grafts. These factors include species of recipient, method of immune deprivation, blood levels of sex hormones in the recipient and size and site of implant.